# Returning to work is not enough for cancer survivors: vocational rehabilitation needs to consider work-related well-being

**DOI:** 10.1007/s11764-026-01998-3

**Published:** 2026-03-10

**Authors:** Katarina Holmberg, Maria Fjell, Linda Eklund, Maria Engström, Angela G. E. M. de Boer, Sven Trygged, Anna Efverman

**Affiliations:** 1 Department of Caring Science, Faculty of Health and Occupational Studies, University of Gävle, Gävle, Sweden; 2 Department of Neurobiology, Care Sciences and Society, Karolinska Institutet, Stockholm, Sweden; 3 Department of Occupational Therapy and Healthcare Counselling, Region Dalarna, Ludvika, Sweden; 4 Center for Clinical Research Dalarna, Uppsala University, Uppsala, Sweden; 5 Amsterdam UMC, University of Amsterdam, Public and Occupational Health, Amsterdam, the Netherlands; 6 Department of Social Work, Criminology and Public Health Sciences, Faculty of Health and Occupational Studies, University of Gävle, Gävle, Sweden

**Keywords:** Cancer survivorship, Vocational rehabilitation, Oncology, Person-centered care, Transition, Return to work, Work-related well-being

## Abstract

**Purpose:**

To explore how cancer survivors experience work-related well-being and aspects influencing their experience of it, one year after cancer treatment.

**Methods:**

During the development of a work-related intervention, cancer survivors (*n* = 22, 67% was female, age md 56 years) were interviewed using purposive sampling one year post chemo-/radiotherapy for breast, prostate or colorectal cancer. Inductive content analysis was applied.

**Results:**

The first year after treatment was described as a transition period in which cancer survivors strove to regain their general well-being but were able to experience work-related well-being even when their general well-being was limited. Their work-related experiences were characterised by a balance between personal readiness for work participation and workload. Daily life and working life were described as interdependent, requiring strategies to maintain balance. There was a vulnerability in handling the demands of work. Both general well-being and work-related well-being were promoted by the absence of side effects and the availability of guidance and responsiveness from caregivers and employers.

**Conclusion:**

One year after treatment, cancer survivors considered participation in work life to be important for their well-being. At the same time, the need for enhanced preparation was expressed for balancing work and private life, managing remaining side effects, and navigating ongoing rehabilitation. Our results suggest that the interplay between personal preparedness and work-related stress is central to work-related well-being, highlighting the importance of addressing work-related aspects early in treatment. The findings also indicate that available resources in cancer rehabilitation are underused relative to patients’ individual needs.

**Implications for Cancer Survivors:**

Cancer survivors desired a more holistic rehabilitation, as both general well-being and work-related well-being encompass life after illness as well as the process of returning to work. Strengthening and clearly defining the role of contact nurses regarding work-related issues, and establishing early collaboration between the cancer survivor, employer, and rehabilitation coordinators, can improve support for return to work. Further research is needed to investigate how these discussions and support efforts can be structured and how models and theories can be applied to contribute to increased focus on issues related to work-related well-being after cancer.

**Supplementary Information:**

The online version contains supplementary material available at https://doi.org/10.1007/s11764-026-01998-3.

## Introduction

A substantial body of research has examined sickness absence among cancer survivors and highlighted the importance of supporting work participation [1, 2]. However, to date, limited attention has been devoted to work-related well-being during the return-to-work process [3], despite evidence that work-related well-being independently was associated with work ability in follow-ups of cancer survivors during long-term survivorship [4]. Accordingly, increased attention to cancer survivors’ work-related well-being is warranted. Well-being is a highly valued, multidimensional concept that includes psychological, social, economic, and philosophical aspects, shaped by subjective needs and cultural context [5]. The World Health Organization defines well-being as ‘*beyond the absence of disease and encompasses a state in which individuals are able to achieve their full physical and mental health potential*’ (WHO, 2025, p.1) [6]. Quantitative measures of cancer survivors’ well-being typically assess subjective perceptions of physical, social, emotional, and functional well-being, which together shape their experiences of health-related quality of life and provide a perspective beyond clinical and objective health measures [7]. This reflects the view that health and well-being are interconnected yet distinct concepts that influence one another while remaining conceptually separate [8] with antecedents related to health and quality of life, such as personal development and meaning [9]. The multidimensional concept of well-being is also applicable to work-related well-being [10], commonly defined as ‘*an individual’s subjective experience and functioning at work*.’ [11]. Work-related well-being may influence general well-being, and vice versa [12]. In this study, we distinguish between the two concepts by referring to the former as work-related well-being and the latter as general well-being. Non-working cancer survivors, one year after completing treatment, reported lower scores on measures of the psychological dimension of general well-being compared with those who had returned to work. As this difference remained significant even after controlling for cancer-specific factors, some researchers consider better general well-being to be a result of re-entering work rather than a precondition for it [13]. Further research on experiences of the interplay between general and work-related well-being is needed.

Work-related factors, such as physically demanding work and work schedules, have been shown to play an important role in cancer survivors’ return to work, together with survivors’ health status, including fatigue and motivation or self-efficacy for work participation [14]. Moreover, limited workplace understanding about various sequelae of cancer treatment and insufficient support influence cancer survivors’ return to work [15, 16]. Work-related well-being has been shown to be important for work ability among long-term cancer survivors, independent of levels of fatigue and work self-efficacy [4] which are otherwise recognized as barriers to work participation and have received considerable attention in recent research [14, 17]. Many cancer survivors do not receive sufficient work-related rehabilitation [18]. When work-related rehabilitation is offered, they primarily aim to facilitate return to work [1, 2], whereas comparatively little attention has been paid to promoting work-related well-being during the return-to-work process [3]. The Geneva Convention [19] stipulates that providers of rehabilitation should ensure general well-being, and the rehabilitation interventions should be of high quality, accessible, delivered by relevant stakeholders, and tailored to the needs of the target population [19]. A lack of continuity and the fragmentation of cancer rehabilitation efforts, such as insufficient attention to work-related well-being, may reduce the long-term effectiveness of work-oriented rehabilitation. These efforts are often not adequately tailored to the individual needs of cancer survivors [20], as healthcare professionals and employers lack knowledge of what cancer survivors consider influencing their experience of work-related well-being (Larsson-Fällman et al., 2026, unpublished data). Accordingly, greater emphasis should be placed on understanding how cancer survivors experience work-related well-being.

In Sweden, healthcare should be person-centered and based on the individual’s participation, needs, and resources [21]. Person-centered care should address the challenges that cancer survivors may experience during the various transition phases of the cancer continuum [22, 23]. By promoting person-centered care, rehabilitation can enhance multiple dimensions of general and work-related well-being and improve overall quality of life, although further research is warranted [24]. When assessing the rehabilitation needs of cancer survivors, issues relating to work-related rehabilitation needs should be included in the assessment, which is primarily carried out by contact nurses and registered nurses [25]. We have previously shown that individuals undergoing cancer treatment have work-related rehabilitation needs early in the course of primary cancer therapy [26]. These needs evolve over time, and the transition from active treatment to the survivorship phase presents distinct challenges [27]. Healthcare professionals and workplace actors face challenges when supporting cancer survivors in the return-to-work process (Larsson-Fällman et al., 2026, unpublished data) [28], often citing limited knowledge and unclear roles and responsibilities as barriers to initiating conversations about work-related needs [29, 30]. To implement effective vocational rehabilitation interventions, it is therefore important to explore cancer survivors’ perspectives on work-related well-being and the factors influencing their experiences, thereby supporting a more sustainable return to work after cancer [3].

This study is guided by the Medical Research Council’s framework for complex interventions, which emphasises that the successful integration of new interventions into routine practice is more likely when the perspectives of the target population are incorporated during the development phase and actively considered throughout the feasibility, evaluation, and implementation phases [31, 32]. In the country where this study was conducted, national guidelines for the first time in 2023 explicitly called for increased efforts in cancer rehabilitation related to work participation [33]. Moreover, in 2021, the screening checklist for cancer rehabilitation needs commonly used in routine care was revised for the first time to include work-related needs [25]. During the fourth phase of the framework [31], the implementation phase, it is important to gain a deeper understanding of cancer survivors’ experiences after these improvements have been integrated into routine practice. These experiences, in turn, inform the development of a new intervention [31] aimed at promoting not only work participation but also work-related well-being one year after cancer treatment, a transitional period that may be particularly demanding [27]. Considering the important role of work-related well-being for work ability, this study aimed to explore how cancer survivors experience work-related well-being and aspects influencing their experience of it, one year after cancer treatment.

## Methods

### Study design

The present study is the second phase (Time-point 2, T2) of a three-phase longitudinal qualitative study in which participants were interviewed while undergoing (T1) [26] and at one (T2) and two (T3) years after curative cancer treatment. A descriptive, inductive qualitative interview design was used.

### Study participants

Study coordinators conducted a purposive inclusion procedure before T1 [26] according to the inclusion criteria cancer survivors, who were of working age between 18 and 64 years, employed or self-employed, when they underwent (neo) adjuvant chemotherapy/radiotherapy for breast, prostate or colorectal cancer in any of the three healthcare regions in the mid Sweden, who possess sufficient proficiency in Swedish to provide informed consent. An exclusion criterion was cancer survivors in a palliative care phase. The previously described purposive inclusion procedure [26] aimed to capture variation across the three selected healthcare regions and the three selected cancer types. The latter was expected to introduce variation in  sex, as breast cancer predominantly affects females and prostate cancer affects males exclusively. By applying purposive sampling criteria, the study aimed to capture variation in additional variables that could influence work-related well-being, such as occupational sector, work demands, and educational level (i.e., secondary school or university education), as these factors differ considerably across the three regions. Two of the regions are traditionally dominated by working-class occupations, such as forestry and industrial jobs, whereas the third region is dominated by white-collar occupations that generally require higher education. Service and educational occupations are present in all three regions. Table 1 presents the self-reported [26] characteristics of the participants and shows that variation was successfully captured with respect to cancer type, healthcare region, occupational sector, demands at work, and educational level but to a lesser extent with respect to sex. At the time for the T2, participants had a median age of 56 years (range 40–65) and were predominantly women, married or cohabiting, who had completed primary cancer treatment for breast, prostate or colorectal cancer about a year ago. At T2, three participants had experienced a cancer recurrence, and four had developed metastases. Less than half, 43%, were undergoing anti-hormonal therapy. All but one had returned to work at least part time, in a variety of occupations with physical or psychological demands. No one had received work-related rehabilitation (Table 1).

**Table 1:** Characteristics of the interviewed participants

Characteristics	Participants, *n* = 22
*Clinical characteristics*	
Cancer type, *n* (%)	
Breast	12 (54)
Prostate	5 (23)
Colorectal	5 (23)
Months since cancer diagnosis^a^, md, 25th–75th percentile, (range)	19, 17–25, (15–102)
Receipt of cancer therapies, *n* (%)	*n* = 22
Chemotherapy^b^	12 (57)
Chemotherapy and radiotherapy	7 (32)
Antihormonal therapy, added to chemo-/radiation	14 (67)
Comorbidities^c^, *n* (%)	8 (36)
Receipt of rehabilitation in general/work-related, *n* (%)	4 (18)/0 (0)
*Sociodemographic characteristics*	
Age in years, md, 25th–75th percentile	56, 50–60
Sex, *n* (%)	
Males	7 (32)
Females	15 (68)
Civil status, *n* (%)	
Married/living with partner	15 (68)
Living alone, have a partner/do not have a partner	3 (14)/4 (18)
Have co-resident children, *n* (%)	8 (36)
Education level, *n* (%)	
Secondary school or occupational education^d^	10 (45)
University	12 (55)
*Work-related characteristics*	
Employment status, *n* (%)	
Employed	21 (95)
Self-employed	1 (5)
Percentages on sickness absence, md, 25th–75th percentile (range)	10, 0–20 (0–100)
Occupational sector	
Education, care or services	10 (46)
Management or entrepreneurship	8 (36)
Technologies or engineering	2 (9)
Other occupations	2 (9)
Demands at work	
At least some physical demands	9^e^ (41)
Psychological demands only	13 (59)

*N*, number and percentages (%) are presented. *Md*, median value

^a^*n* = 2 had cancer for the second time; time since the first diagnosis was presented

^b^*n* = 1 was still undergoing chemotherapy at the time of the interview due to recurrence

^c^Long-term pain, cardiovascular conditions, and mental health conditions were reported

^d^None had only elementary education (according to Statistics Sweden, only approximately 10% of the working-age population have only elementary education (34)). Occupational education was not at university level

^e^Two reported exclusively physical demands. The distribution across the healthcare regions was = 6, *n* = 9, and *n* = 7

### Data collection

An occupational therapist, L.E. (MsC), and a Registered nurse, M.F (PhD), interviewed the cancer survivors individually [35] regarding how they experience work-related well-being and aspects influencing their experience of it. Both interviewers have professional clinical experience in cancer care and prior experience in conducting qualitative interviews. This facilitated their understanding of the participants’ experiences and enabled them to ask relevant follow-up questions while adhering to the semi-structured interview guide used in all interviews (Table 2). The study’s purpose guided the development of the interview guide, which was prepared prior to T1 to ensure that key aspects of work in relation to cancer, as outlined in a conceptual model [36], were explored through open-ended questions, supplemented by follow-up questions when needed. The interviewers conducted and recorded the interviews either digitally (approximately 75%) or at a location chosen by the participants, ensuring privacy. To maintain confidentiality [37], the participant’s name was replaced with a code number during the interview and when transcribed verbatim. Fifty percent of the interviews lasted longer than 30 min (median), 25 percent lasted less than 23 min, and 75 percent lasted longer than 40 min.

**Table 2:** The semi-structured interview guide

Interview questions
1. Can you describe your current work situation?^a^
2. How do you feel your well-being is affected based on your current work situation?^a^
3. What facilitates or hinders your work situation?
4.What are your thoughts about your chance of being able to return to/stay in work^b^ in the long run?
5. Can you describe the support you experience you are receiving currently, related to your well-being in your current work situation?^a^

The interviewer began by introducing herself. General follow-up questions: ‘Can you tell me more?’; ‘What do you mean?’ Follow-up questions concerned what facilitates and what hinders work participation and work-related well-being

^a^Regardless of whether the cancer survivor had returned to work or was on sick leave

^b^The formulation was adapted depending on the current work situation. When necessary, field notes were made

The study was performed in line with the principles of the Declaration of Helsinki [38]. Approval was granted by the Swedish Ethical Review Authority (2022–03686-01), and the study was reported in accordance with the Consolidated Criteria for Reporting Qualitative Research (COREQ) [39]. All participants gave written consent at T1 and oral telephone consent before T2, after being informed of the study was voluntary and unrelated to care and employment.

### Data analysis

The first (KH) and the last author (AE) are registered healthcare professionals (nurse and physiotherapist, respectively), both female, with prior experience in cancer care and in conducting qualitative research. They analyzed the interview data using content analysis as described by Graneheim and Lundman [40]. The analyzing authors read the transcripts several times to gain an overall understanding of the content of the interview data [40]. The meaning units in the transcripts were identified and initially organized into three clearly identifiable content areas: well-being, work issues and social issues, which were marked with different colors representing the main topics in the interview guide in relation to the study aim, thus providing a preliminary structure for the analysis, as previously suggested [40]. KH condensed the colour-coded meaning units and labelled them with descriptions under each content area. During this process, some codes were merged when they described similar content or overlapped across content areas, while others were reworded for clarity and to better reflect the meaning of the content. After KH and AE had reviewed and discussed all codes, KH identified patterns and subcategories, and formulated categories as part of the manifest phase of the analysis [40]; see Table 3. Graneheim and Lundman [40] describe categories as the manifest part of the data analyses, “what” the participants talk about. When the subcategories and categories had been developed, the last author (AE) reread the transcripts and reviewed the codes and categories.

**Table 3:** Examples of the data analysis process

Meaning unit	Condensed meaning unit	Code	Sub-category	Category
I think it’s fun to work, I feel… well, important and meaningful, and I don't want to be sick [laughs], I want to be healthy, and I want to have energy. You support yourself with your own money, it was really nice when I broke contact with… or when I didn't need to be on sick leave anymore. Then I was healthy	It’s fun to work, I feel important and meaningful. I want to be healthy, and I want to have energy… I support myself with my own money, it was nice when I didn’t have to be on sick leave anymore. Then I was healthy	A feeling of strength A desire for normality Autonomy	Reconnecting life and work	The relationship between well-being and work-related well-being
Of course I’m more tired… You’re not really there. It’s a kind of tiredness that’s hard to put your finger on. You can say you’ve been tired before, but this is a different kind of tiredness that you can't just lie down and rest away. It almost helps more… it’s better if I go out for a walk to clear my head than to lie down and rest	I’m more tired… You can say you’ve been tired before, but this is a different kind of tiredness that you can’t just lie down and rest away… it’s better if I go out for a walk to clear my head than to lie down and rest	Fatigue	Vulnerability to work demands	The dynamics of readiness and work strain in the interplay between well-being and work-related well-being

The principle for selecting meaning units from the content of the interviews and condensing the meaning units is presented, exemplifying the link between them and the codes, sub-categories and categories

Following these steps in the content analysis, one author (ST), who had not previously participated in the interviews or the analysis, independently read all transcripts and reviewed the categories in relation to the original data. This was followed by a discussion of the findings with the authors who had participated in the analysis. This approach aimed to ensure that the themes, categories, and subcategories accurately reflected the participants’ perceptions and to enhance the credibility of the analysis [41]. Further discussions were then held within the full author team (6 PhDs, 1 MsC), which has diverse professional backgrounds in healthcare and social services. These discussions aimed to critically reflect on emerging interpretations, ensure that the analysis remained grounded in the participants’ perspectives, and promote transparency throughout the process, in line with methodological recommendations [40]. Minor adjustments were subsequently made, resulting in the final analysis.

## Results

**Fig. 1:**
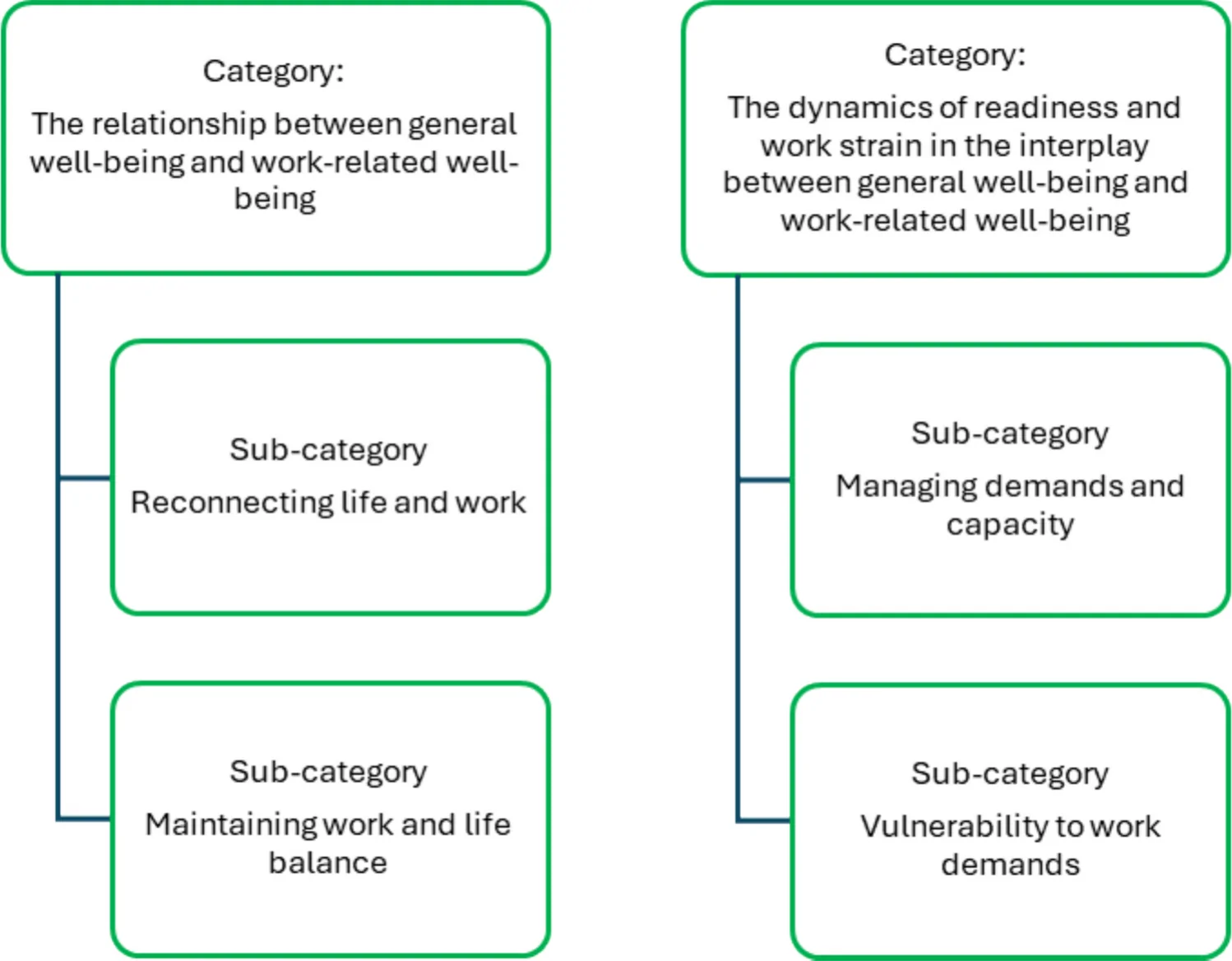
Schematic presentation of the results of the content analysis, comprising two categories, each with two subcategories

Cancer survivors’ experiences of work-related well-being and the factors influencing it are described in two categories: (1) the relationship between general well-being and work-related well-being, and (2) the dynamics of readiness and work strain in the interplay between general and work-related well-being. Each category comprises two subcategories and is presented in Fig. 1. Our results indicate that experiences of general well-being after cancer treatment influence work-related well-being. During the first year after treatment, a critical transition period, cancer survivors actively pursued general well-being, and work-related well-being could at times be experienced despite ongoing challenges to general health. Personal readiness and work-related demands shaped and tested these experiences. Survivors described a reciprocal relationship between life and work that required conscious strategies to maintain balance. The absence of treatment-related side effects, together with guidance and responsiveness, promoted general well-being, whereas the lack of these factors disrupted both general and work-related well-being.

*“Some days are spot on. Everything goes smoothly; I have energy. I manage what I need to do, I come home, I take care of things at home and so on. And then there are days like today, where I`m like a wet rag. It hurts in strange places. I feel nauseous. I´m just so tired, it makes me anxious”.*

### The relationship between general well-being and work-related well-being

This category highlights the importance of connecting with life and work and maintaining a balance between the two to support overall well-being and well-being related to work.

#### Reconnecting life and work

Participants described work as a lifeline after treatment. Their experiences reflected efforts to re-establish daily routines, where control over everyday life contributed to general well-being. The importance of being independent and contributing to society through work was described as a source of meaning and social function. Returning to work contributed to work-related well-being, despite ongoing challenges to general well-being such as fatigue, anxiety or financial worries.

*“I enjoy my work, so in that sense it has been nice to get back to work. It’s a bit more normal, so to speak. When you’re at home on sick leave and not feeling well, either physically or mentally, the walls start to close in on you.”*

Participants felt optimistic about returning to work and everyday life, resuming familiar roles and activities that provided a sense of purpose. Regaining independence and financial stability after a period of reliance on others and sickness benefits was highly valued. At the same time this optimism was tempered by concerns that returning to work might be premature and that more emphasis should have been placed on recovery.

*“I’m a bit torn right now, whether I should go on sick leave again because of the fatigue, since it’s like… because it’s as if… Resting on the days in between work doesn’t really help.”*

Illness and treatment could also act as a catalyst for change, prompting participants to reduce working hours or changing jobs to prioritize their well-being. They also felt a personal responsibility to support a smooth return to work in collaboration with their employers.

#### Maintaining work and life balance

Participants experienced recovery through physical activity, rest, walks or mind–body exercises such as mindfulness as personal necessities for well-being and emotional balance. Recovery reduced fatigue and provided support in life as well as working life.

*“… before the actual treatment they recommend that you start exercising. And I took that on board and have done that. And then I discovered myself that I felt much better when exercising, so that was good.”*

When routines were disrupted by physical limitations or lack of time, participants reported a decline in well-being. Recovery was often perceived as a lengthy process, with participants struggling to adapt to and accept a slower pace of life. Challenges arose from difficulty recognizing the need for rest and balancing external responsibilities such as work, family, and social life. As a result, important recovery activities were often postponed to conserve energy for work.

### The dynamics of readiness and work strain in the interplay between general well-being and work-related well-being

This category encompasses perceptions of the complex interplay between individuals’ readiness to return to work and how internal and external work challenges can affect well-being in both positive and negative ways.

#### Managing demands and capacity

During the first year after cancer treatment, participants emphasized the importance of receiving guidance, adaptation, and understanding from others to support sustainable recovery and well-being. The participants wanted an open and transparent dialogue with their employers, as they considered it important for well-being in the workplace. However, they perceived discussions with employers as brief and informal. Health remained a source of concern, and participants expressed a desire for information from healthcare providers not only about somatic issues but also about their daily functioning and working life. Although unanswered questions about health and work did not directly affect their ability to perform tasks, they contributed to anxiety and worry, reducing overall well-being.

*“If you get answers, you might have certain things that help you feel calmer. Then it’s easier both to work and to go on living. That’s something I would have liked, especially when you’re quite young and have a lot left in life, and then it’s not so good to have a lot of worry.”*

The process of balancing work, health and social life was described as energy-consuming and requiring advice and support to manage both pitfalls and overconfidence in one’s own abilities.

*“... I don’t know, it was like the whole world came crashing down on me and I just became flat. And no energy, no desire, pain, and feeling sick. My body is just exhausted. And it’s a setback... I want to move forward.”*

Active involvement in work planning and scheduling proved helpful. Involvement gave individuals a sense of control over their work situation and thereby increased work-related well-being. Involvement also facilitated consideration of poorer overall well-being during the workday, for example after a poor night’s sleep.

*“I have some flexibility and can start a bit later in the morning if I don’t have any scheduled appointments or someone waiting for me.”*

A flexible and supportive employer attitude, such as meeting employees’ physical well-being needs, e.g., access to toilets when suffering from urgency or leakage, or offering remote work and flexible hours, was described as promoting work-related well-being. Having opportunities to engage in physical activity or body-mind practices during working hours was considered beneficial, as was having access to a private space at work that allowed withdrawal and reduced sensory input thus enabling cognitive rest.

#### Vulnerability to work demands

Side effects were reported to influence recovery, health and work performance, preventing participants from reaching their full potential. Despite the need for support often being identified early in treatment, it was rarely addressed during recovery. Participants expressed a desire for regular follow-ups, as their well-being fluctuated with changes in physical, mental, and psychosocial conditions.

*“I have questions that pop up all the time... And if you know you might have a doctor’s appointment... It could just be a quarter of an hour... that they call you, if you have a few questions, because that’s what I would have liked, maybe every three months during the first year, just a quarter of an hour, to.../…/And if you have questions, you get answers to them instead of... Now it’s all questions... I don’t get answers to them*.”

The participants experienced a lack of information regarding treatment-related side effects and limited opportunities to discuss physical changes and their impact on body image. Treatments such as hormone therapy and surgical castration were described as affecting perceptions of identity and social roles, contributing to reduced well-being, including low mood and, in some cases, depression. Counselling was recognized as an important aspect of care, but was often experienced as insufficient, in that practical concerns were prioritized over psychological and existential support.

*“This treatment has hit me harder than I expected. I’m on testosterone therapy, and it hasn’t been as gentle as I thought it would be. It wears you down, little by little. One thing leads to another. I was completely symptom-free during the first six months, but then it started. My mind began to waver.”*

Fatigue was described as consuming nearly all energy at work, with recovery during evenings and weekends rarely being sufficient. Susceptibility to infection following treatment caused anxiety when work required presence at the workplace. Participants who were able to work from home, mainly white-collar workers and people in management positions, reported positive effects on their well-being due to reduced exposure to circulating viruses. The ability to delegate or redistribute work tasks was considered beneficial, as it minimized the need for physical attendance. Working on site was experienced as particularly stressful, not only because of increased exposure to viruses, but also because of changes in routines. Changed schedules, travel, different food and limited access to facilities such as toilets and rest areas had a negative impact on general well-being and affected work-related well-being. Participants employed in the service sector had fewer opportunities for breaks and restorative activities during working hours and were required to be on site with fixed schedules.

*“I try to do the things that are recommended, like trying to go out for a walk or rest if I have the opportunity and if I’m at home. If I’m at work, then that’s just how it is. I work and then I get to rest later.”*

Ensuring well-being in the workplace required cooperation between employers, healthcare providers, and employees, which helped reduce vulnerability during return to working life.

## Discussion

One year after cancer treatment, the survivors interviewed in this study experienced work participation as important for both general and work-related well-being, yet they often feel unprepared to balance work with personal life, lingering side effects, and recovery activities. They described general well-being as closely linked to work-related well-being, though some reported experiencing good work-related well-being despite poor general well-being. The dynamic interplay between personal readiness and work-related strain was perceived to influence work-related well-being within this relationship.

These findings clearly indicate that for most cancer survivors, simply returning to work is not enough; they also desire work-oriented support that considers their work-related well-being. Previous research has shown that returning to everyday life is an important part of recovery during the first year after cancer treatment [42], and that work serves as a key anchor for reclaiming one’s identity during this period [26]. However, the current findings suggest that returning to work may jeopardize several dimensions of overall well-being if the balance between personal readiness and work-related demands does not adequately meet the needs of cancer survivors. Such experiences are common during the period after the end of treatment, known as the re-entry phase [43], and are associated with changes in physical and emotional functioning, interpersonal relationships and personal outlook on life after cancer treatment [43]. In the current study, concrete work tasks helped to alleviate the psychological stress that sometimes followed treatment. In this way, returning to work contributed to work-related well-being, even when various aspects of general well-being were not fully experienced.

The experiences of interviewed cancer survivors regarding work-related well-being should be understood in the light of their expectations, which truly were shaped by their own and others’ past experiences with illness [44]. A common phenomenon in the re-entry phase is the expectation of quickly resuming the roles held prior to a cancer diagnosis [43]. This expectation was also evident in the current study, as participants often anticipated that life would continue as before. Although this rapid return to former life may reflect participants’ desire to return to normal, it also suggests a potential overestimation of their perceived readiness to cope with the physical, emotional, and cognitive demands of everyday life after treatment. Such assumptions about readiness may obscure the strain associated with balancing recovery alongside pre-illness responsibilities [43], thereby increasing the risk of setbacks. The pressure to resume previous routines may stem not only from personal motivation but also from social, economic [45], and professional expectations. Such pressure risks create a mismatch between the survivor’s functional capacity and work demands, where the desire to work may exceed actual ability [36], potentially compromising both overall and work-related well-being.

For the study participants, health remained an important focus, and they expressed a desire for information from healthcare providers not only about physical health but also about their daily functioning and work life. By identifying contact nurses as an important but underused resource for managing both well-being and work-related well-being among survivors, the results highlight a concrete way to integrate professional and everyday perspectives into cancer rehabilitation in an already existing professional group. Contact nurses can help shift the focus from purely medical recovery to sustainable participation in working life by more systematically raising these issues in patient encounters. To support this focus, the Cancer and Work Model [36] can be helpful, as it was developed for work-related rehabilitation after a cancer diagnosis. By applying the model, symptoms, function and work environment, areas that are central to the patient's recovery and well-being, can be strengthened. Furthermore, collaboration between contact nurses and professional rehabilitation coordinators needs to be increased. The role of the rehabilitation coordinator is specifically to coordinate rehabilitation needs and organize interventions to facilitate a return to work during sick leave, as well as to provide support to both physicians and external stakeholders (Lag om koordineringsinsatser för sjukskrivna patienter [Coordination Act for Sick-Listed Patients], 2019) [46]. Based on the findings of the current study, the role of rehabilitation coordinators in cancer rehabilitation appears to be limited.

When exploring factors influencing the experience of work-related well-being, we aimed to capture each interviewed cancer survivor’s individual perspective. As the definition of the concept underscores its subjective nature [10, 11], participants were encouraged to describe it in their own terms. The currently interviewed cancer survivors experienced joy in returning to work while also facing stress related to challenges during recovery. For example, participants emphasized that engaging in physical activity contributed to their general well-being, which in turn supported their work-related well-being and work ability, consistent with evidence from research [2]. However, finding the energy to engage in physical activity was not easy. Cancer-related fatigue and limited workplace flexibility were described as barriers to engaging in physical activity. As with other long-term illnesses, opportunities for general and work-related well-being still appear to depend on social factors [47]. Participants working in the service and care sector particularly expressed these challenges, whereas white-collar participants had greater flexibility to influence their work, leading to more opportunities for restorative activities. Previous research on long-term health issues in both cancer [1, 2] and non-cancer populations [48] has identified flexibility as an important factor for work participation, whereas less is known about its effects on general or work-related well-being. During active cancer treatment, participants sometimes experienced that workplace flexibility posed a threat to their general well-being, as they would otherwise have needed to take full sick leave to rest and recover [26]. One year later, however, flexibility was perceived exclusively as beneficial.

When discussing the actions and actors perceived to influence work-related well-being, cancer survivors identified healthcare professionals, employers, and family members as key stakeholders whose actions and understanding could either support or undermine their work-related well-being. In a previous interview study, cancer survivors reported receiving support during the transition back to work primarily from family and friends, along with some pressure from the physician who issued the sickness certificate [49]. Existing evidence suggests that both healthcare professionals and workplace actors face challenges in addressing the work-related rehabilitation needs of cancer survivors (Larsson-Fällman et al., 2026, unpublished data) [28]. Limited knowledge and unclear roles and responsibilities [29, 30] can hinder discussions about work and create barriers to providing appropriate support. Employees with long-term illness may avoid disclosing their condition or need for accommodations due to fear of stigma, negative judgments, or being seen as a burden [50]. On the other hand, employers often report difficulties in understanding survivors’ needs and may struggle to initiate work-related conversations after treatment, further limiting the support available to returning employees (Larsson-Fällman et al., 2026, unpublished data) [51]. Awareness of and preparedness for the physical, mental, emotional, and social vulnerabilities that often follow a cancer diagnosis can help facilitate a sustainable transition back to working life [52]. In this context, a person-centered dialogue allows support to be tailored to the survivor’s individual needs [53] in the certain work environment [48]. In Sweden, a checklist for rehabilitation needs [25] is recommended for use in cancer care. The checklist is primarily used by contact nurses to identify patients’ rehabilitation needs and tailor support, with one of its 19 questions specifically addressing work [25]. However, study results showed that participants rarely had the opportunity to discuss work or return to work, as the focus was primarily on somatic conditions. In this context, the initiative is aimed at contact nurses, who meet and regularly follow up with patients and thus have a key role in also asking about work-related needs.

### Methodological considerations

The purposive sampling [26] captured variation in cancer type, cancer treatment, healthcare region, occupational sector, demands at work, and educational level, but to a lesser extent with respect to  sex, as two-thirds of the participants were female. This was not entirely unexpected, as one of the three included cancer types was breast cancer, which affects almost exclusively females. We sought representation of both sexes among participants with colorectal cancer, which occurs in both men and women. However, men with prostate cancer did not offset the predominance of female participants. Some evidence indicates that women are more likely than men to take part in health research studies [54]. One limitation of our study is that none of the participants had only an elementary school education. Despite efforts to recruit cancer survivors from diverse educational backgrounds, we were unable to include individuals with only primary school education. This was not unexpected, as they constitute approximately 10 percent of the Swedish population, the majority of whom are immigrants from developing countries [34]. Additionally, one of the study criteria was sufficient proficiency in Swedish to provide informed consent and participate in an interview without an interpreter. Including participants who had received different cancer treatments (neo- or adjuvant chemotherapy and/or radiotherapy) resulted in variation in time since diagnosis, as chemotherapy typically extends over a longer period than radiotherapy. Two outliers had an exceptionally long time since diagnosis due to extensive treatment; excluding these, time since diagnosis ranged from 15 to 35 months. The diversity of participants regarding important variables and their subjective experience strengthened the credibility of the findings by providing multiple in-depth perspectives [55] on work-related well-being. Recruiting participants during ongoing cancer treatment enabled the inclusion of diverse experiences of work-related well-being and reduced the risk of overrepresenting cancer survivors with problematic return-to-work experiences.

As suggested for qualitative interview studies [35], the richness and adequacy of data are more important to consider than the number of interviewed participants [56]. Given the purposive sampling of 22 participants and the interview method discussed below, we obtained a rich dataset. All participants had previously been interviewed regarding work-related issues [26], which may have contributed to increased awareness of work-related well-being compared to other cancer survivors. While this continuity enriched the data by providing deeper insights, it should also be recognized as influencing participants’ experiences compared to possible interview data from naive cancer survivors. Naturally, all experiences shape future experiences, and in line with qualitative research principles, the study drew on the participants’ experiences. The interviewers were experienced in cancer care and rehabilitation, which facilitated a deeper understanding of the participants’ experiences. They used a semi-structured interview guide [35] to enhance dependability while allowing the cancer survivors to describe their experiences freely. As suggested [35], the interviewer encouraged the descriptions without being overly directive, while none-the-less capturing all aspects of the study aim. The authors primarily involved in the analysis supported credibility by a structured analytical process in which they repeatedly discussed the analysis to ensure the consistency and depth of the interpretation. Furthermore, an author who so far had not been involved neither in the analysis process, nor in the interviews, reviewed the content of the interview transcripts and its alignment with the developed categories and subcategories to support credibility [41]. The categories and subcategories were critically discussed within the entire author team from a methodological perspective, thereby enhancing the transparency and confirmability of the analysis. Exemplifying each presented category with quotes further enhanced these quality aspects [40]. To reduce participant burden, we did not request that participants review the transcripts or data analysis. We reported the study in accordance with COREQ [39], which strengthened transparency when presenting the research team, study participants and setting, data collection and analysis, and the presentation of interpretations and results. Providing detailed descriptive information on participants’ sociodemographic, clinical, and work-related characteristics enables readers to assess the population to which the findings are applicable and transparently demonstrates the predominance of relatively highly educated women. As in many other countries with generous social insurance systems, where society rather than employers or employees’ bears the costs of sickness absence, work-related rehabilitation remains underdeveloped in Sweden, the country examined in this study. None of the participants had received any related rehabilitation, and only 18% had received any form of rehabilitation overall, despite several still being on at least part-time sick leave and experiencing side effects from cancer treatment. As with all qualitative interview studies [35], the transferability to other populations is limited; the study contributed to deeper insights into cancer survivors’ experiences in the development phase of a work-oriented intervention [31, 32] and did not quantify generalizable data.

### Implications for cancer survivors

Cancer survivors desired a more holistic rehabilitation, as both general well-being and work-related well-being encompass life after illness as well as the process of returning to work. Strengthening and clearly defining the role of contact nurses regarding work-related issues, and establishing early collaboration between the cancer survivor, employer, and rehabilitation coordinators, can improve support for return to work. Further research is needed to investigate how these discussions and support efforts can be structured and how models and theories can be applied to contribute to increased focus on issues related to workrelated well-being after cancer.

## Conclusion

One year after treatment, cancer survivors considered participation in work life to be important for their well-being. At the same time, the need for enhanced preparation was expressed for balancing work and private life, managing remaining side effects, and navigating ongoing rehabilitation. Our results suggest that the interplay between personal preparedness and work-related stress is central to work-related well-being, highlighting the importance of addressing work-related aspects early in treatment. The findings also indicate that available resources in cancer rehabilitation are underused relative to patients’ individual needs.

## Supplementary Information

Below is the link to the electronic supplementary material.

Supplementary file1 (DOCX 29.8 KB)

## Data Availability

The data generated from this study are not shared openly to preserve individuals´ privacy under the European General Data Protection Regulation; confidential data might be available upon request to the corresponding author.
